# Adequate antenatal care service utilizations after the onset of COVID-19 pandemic in Ethiopia: a systematic review and meta-analysis

**DOI:** 10.3389/fpubh.2024.1395190

**Published:** 2024-11-15

**Authors:** Temesgen Gebeyehu Wondmeneh, Zelalem Solomon Tadesse

**Affiliations:** ^1^Department of Public Health, College of Medical and Health Science, Samara University, Semera, Ethiopia; ^2^Department of Management, College of Business and Economics, Samara University, Semera, Ethiopia

**Keywords:** adequate, antenatal care, service, utilization, Ethiopia

## Abstract

**Background:**

The world faces great difficulty in continuing to provide essential maternity health care after the onset of COVID-19 pandemic Many women have trouble accessing maternity healthcare due to fear of infection. A decline in the utilization of maternity health services is suggested to worsen adequate antenatal care service utilization. Thus, this study aimed to determine the pooled estimate of adequate antenatal care service utilization after the onset of COVID-19 in Ethiopia.

**Methods:**

The searching of articles was carried out on Web of Science, Scopus, PubMed, CINHAL, Google Scholar, African journals online, and the institutional repository of Ethiopian universities. Using a Microsoft Excel standardized spreadsheet, the data were extracted. A random effect model was used to determine a pooled estimate of adequate antenatal care utilization. *I*^2^ statistics were used to quantify the amount of heterogeneity. The evidence of publication bias was examined using Egger's regression test and a visual inspection of the funnel plot. Subgroup and sensitivity analyses were also carried out.

**Results:**

Finally, this systematic review and meta-analysis included 11 eligible articles. The overall pooled estimate of adequate antenatal care service utilization after the onset of COVID-19 pandemic in Ethiopia was 46.28% (95% CI: 35.32%−57.26%). There is a substantial amount of heterogeneity between studies (*I*^2^ = 99.07%, *p* < 0.001). Pregnant women who visited antenatal care early were 10.9 times more likely to have adequate antenatal care utilization than those without early visits (AOR = 10.93, 95% CI: 7.2–14.66).

**Conclusion:**

In this review, the percentage of women who utilized adequate antenatal care after the onset of COVID-19 pandemic in Ethiopia was less than half. Early antenatal care visit is an important factor to achieve adequate antenatal care service utilizations.

**Systematic review registration:**

: CRD42023495279.

## Introduction

Antenatal care (ANC) is an essential component of improving maternal and newborn health (1). For all pregnant women, WHO recommends at least four ANC visits (2). In Ethiopia, primary healthcare centers provided health services such as maternal and child healthcare, education on health problems, the development of effective food supply and proper nutrition, safe water supply and basic sanitation, endemic disease control, treatment of common diseases and injuries, and provision of essential medications. Community health extension workers have significantly contributed to the increase in women's utilization of ANC, family planning, and HIV testing (3, 4). Globally, COVID-19 pandemic has caused substantial disruptions to issues related to politics, health, economy, and society (5). The COVID-19 pandemic affected the provision of all health care services globally (6). Health care systems have been affected by the COVID-19 pandemic in many ways, such as the temporary closure of services (7) and the utilization of non-COVID services (8). The COVID-19 pandemic has caused major issues for pregnant women, as hospitals and healthcare providers have found it difficult to address the demands of infection prevention, pandemic preparedness, treating a high number of critically ill patients, and attending to the needs of non-urgent pregnant patients (9). Due to COVID-19 pandemic-related restrictions, there may be decreased perceived social support and limited access to prenatal services, which could exacerbate pregnancy-specific stress (10). The findings of the literature review showed that health systems, especially in Sub-Saharan Africa, have concerns about the necessity of giving women's ANC priority during COVID-19 pandemic in order to ensure the safety of the newborn baby (11).

A systematic review and meta-analysis study done globally revealed that ANC appointments decreased by 38.6% throughout the pandemic period (12). During COVID-19's second wave, about 39.5% of pregnant women in Indonesia did not receive ANC services (13). In Brazil, ANC was considered adequate for 35.8% of postpartum women (14). During COVID-19 pandemic, 46.4% of Filipino women received adequate ANC (15). Adequate ANC service utilization was 12.7% during COVID-19 pandemic in Chiapas (16). In a study of sub-Saharan Africa, the country's median reduction in utilization of health services in 2020 was 3.9%. The highest reductions were observed for inpatient and outpatient admissions, while ANC, delivery care, and immunization services had smaller reductions. Eastern African countries had the highest reductions (17). In a hospital-based study in Georgia, there were 965 women who accessed prenatal care prior to COVID-19 pandemic and 793 women accessed during the pandemic. The mean number of prenatal care visits was almost comparable prior to COVID-19 and during the COVID-19 pandemic (mean = 6.9 prior to the pandemic vs. 7.1 after the pandemic) (18). In a study of four Sub-Saharan countries (Benin, Malawi, Tanzania, and Uganda), the number of ANC visits during the pandemic (2020) and before the pandemic (2019) was comparable; however, decreases in the number of consultations were noted in Tanzania, Malawi, and Uganda in 2020 compared to 2019 (19). Demographic and Health Surveys (DHS) data from 2010 to 2018 showed that the optimal ANC utilization rate in East African nations was 56.37%, with Rwanda having the lowest optimal ANC utilization (44.3%) and Zimbabwe having the highest optimal ANC utilization (81%) (20). The pandemic caused the ANC's level of adequacy to drop by 75.8% compared with the pre-pandemic rate (16). During the COVID-19 pandemic in 2020, Uganda had ANC service attendance rate of 48.2%. There was a 7.1% reduction in the number of ANC visits during the COVID-19 lockdown period, compared to prior to the COVID-19 pandemic in 2019 (21). In Kenya, about 20% of women before the pandemic achieved adequate ANC utilization, whereas 14% of women achieved adequate ANC utilization during the COVID-19 pandemic (22).

A systematic review conducted in Ethiopia showed that the pooled reduction of ANC was 19.3%, family planning was 26.6%, institutional delivery was 12.8%, postnatal care was 17.8%, and abortion care was 19.4% (23). Using mini-EDHS 2019, 43% of women had optimal ANC visits during their last pregnancy (24). A study at the district level in Ethiopia conducted between 2019 and 2020 indicated that 43.7% of women in their last birth received four or more ANC (25). In Ethiopia, studies reporting the proportion of ANC utilization before the COVID-10 pandemic (2018–2019) and after the COVID pandemic (2020–2021) are provided in Table 1.

**Table 1:** The proportion of ANC service utilization before the COVID-19 pandemic and after the COVID-19 pandemic.

**Study site**	**Before COVID-19 (2018–2019) proportion of ANC utilization**	**After COVID-19 (2020–2021) proportion of ANC utilization**	**References**
Overall proportion of ANC utilization	60.3%	61.85%	(26)
Amhara region	56.6%	55.4%	
Oromia region	64%	62.6%	
Dire Dawa	41.4%	43.8%	
SNNP	85.3%	79.7%	
Afar	52.7%	48.5%	
Sidama	64.3%	80.8%	
Dire Dawa administrative town	46.2%	53.8%	(27)

SNNP, Southern Nation Nationality People.

The fear of contracting COVID-19, anxiety, feeling vulnerable, the desire to protect oneself and loved ones, the stay-at-home policy, transportation issues, limited social interaction, high-risk pregnancies, a lack of knowledge, and adherence to COVID-19 preventive behaviors were obstacles to accessing ANC during COVID-19 pandemic (28). The COVID-19 pandemic had significant morbidity and socioeconomic impacts associated with receiving low-quality and accessing maternity healthcare as a result of fear of infection and the use of containment strategies such as social distance and community restriction (29). Government COVID-19 movement restrictions and the subsequent limited transport access resulted in a high cost of transport, which played a role in reducing mothers' access to ANC services (30). A facility-based study conducted in Ethiopia found a reduction in receiving recommended prenatal care services due to fear of COVID-19 and service interruptions caused by the pandemic (31). A community-based cross-sectional study in Ethiopia revealed that distance to health facilities was a challenge to accessing maternal health care service utilization amid the COVID-19 pandemic (32). In Ethiopia, community-based health insurance improves health service utilization (33, 34). Recommended ANC utilization was higher among mothers with health insurance than those with no health insurance (35, 36).

Improving health system resilience through integrated health service delivery is a potential strategy during the outbreak of the disease (37). There is no reason to worry about maternal and child health services being overshadowed by COVID-19 (38). Proper prenatal care is essential for the health of the pregnant mother and the developing baby. Inadequate prenatal care causes low birth weight, preterm birth, and miscarriage (39). Adequate antenatal care service utilization is essential to both mother and child health; however, many studies have reported that COVID-19 pandemics affect many provisions of health service delivery, including ANC service, especially in developing countries. Even though there were district-level studies about adequate ANC service utilization in Ethiopia after the onset of COVID-19 pandemic, there is a lack of integrated data regarding the use of adequate ANC service utilization after the onset of the COVID-19 pandemic at the national level. Therefore, the aim of this systematic review and meta-analysis was to determine the pooled magnitude of adequate ANC service utilization after the onset of COVID-19 pandemic in Ethiopia.

## Methods

### Protocol and registration

The Preferred Reporting Items for Systematic Reviews and Meta-Analyses (PRISMA-2020) checklist (40) (Supplementary File S1) was followed in the reporting of this systematic review and meta-analysis. The systematic review was registered on the International Prospective Register of Systematic Reviews (PROSPERO) with identification number CRD42023495279.

### Search strategies

Comprehensive search strategies were conducted from February 15, 2024, to February 22, 2024, using distinct electronic databases such as PubMed, CINHAL, Google Scholar, and African journals online. To find studies that were missed by electronic database searches, further manual searches were conducted using online higher education repositories, government institutes, and reference lists. The following keywords served as the basis for the search strings or terms: adequate, antenatal care, and Ethiopia. Using the Boolean operator “AND,” the keywords “adequate,” “antenatal care,” and “Ethiopia” were combined. The “OR” Boolean operator was used to combine synonyms for each of the key search terms. The search was restricted to studies with full text, published in the English language, and a publication date between January 1, 2020, and February 22, 2024, where a database could allow this filter. Further information regarding the search strategy of the selected databases is attached (Supplementary File S2).

### Study selection

Two authors (TGW and ZS) carried out the search strategies from the relevant databases. Studies identified through different database searches were exported to Endnote X8.1 software. Duplicated studies were removed by Endnote X8.1 software. TGW and ZS independently screened the selected articles for the relevance of the review objective based on the titles and abstracts. After the screening of titles and abstracts, the full texts of articles considered relevant were retrieved. The eligibility of each article was assessed independently by two authors (TGW and ZS) after reviewing their full texts. Scientific consensus settled the disagreements between the two authors.

### Eligible criteria

To establish the inclusion and exclusion criteria for this systematic review and meta-analysis, the population, intervention, comparator, and outcome (PICO) framework technique was used, which mainly involves population, outcome, and context (POCo) questions for prevalence studies. The inclusion and exclusion criteria were presented in Table 2.

**Table 2:** Inclusion and exclusion criteria.

**Inclusion criteria**	**Exclusion criteria**
The study population consisted of reproductive-age women who gave birth at least once	Qualitative studies, reviews, commentary, and editorials
Adequate ANC was the number of ANC coverage of at least four or above during women's pregnancy reported in the primary studies in accordance with the definition (41, 42)	Only outcomes reported on delayed initiation of ANC and quality of ANC
	Studies on pregnant women who were still receiving ANC follow-up or who had not completed their ANC follow-up
Studies conducted in Ethiopia	Studies on national levels, such as EDHS
Studies conducted from 01/01/2020 to 02/22/2024 were included	Studies that failed to provide definitive information about adequate ANC
Both descriptive and observational study designs reporting the magnitude of adequate ANC (at least four or more ANC follow-ups during pregnancy)	
Published and unpublished full-text articles	
Only studies published in English were reviewed	

### Operational definition

The number of ANC coverage of at least four or above during women's pregnancy reported in the primary studies was considered adequate ANC; otherwise, it is inadequate ANC (41, 42).

### Data extraction process

Relevant data from the primary papers was extracted independently by two authors (TGW and ZS). A standardized data extraction format created from Microsoft Excel was used to retrieve the data. For each included study, the following information was extracted: the first author's name, publication year, the study period, the study region, the study setting, the study subjects, the sample size, the number of cases with adequate ANC, and the proportion of adequate ANC. A scientific consensus was used to resolve the disagreements between the two authors. Data for the associated factors were retrieved, and the odds ratio for each factor was extracted using two by two tables (Supplementary File S3).

### Quality assessment

The Newcastle-Ottawa Scale for evaluation of the quality of the cohort is adapted to assess the quality of the included studies in this systematic review and meta-analysis (43). Two authors (TGW and ZS) independently assessed the qualities of the included studies. Subjectivities between the two authors were settled by using the mean score of the reviewers' results as well as through scientific consensus. The qualities of the included studies were evaluated using ten-star ratings in three domains. The first is selection (five stars): sample representativeness (one star), sampling technique (one star), response rate (one star), and exposure ascertainment (two stars). The second is comparability (two stars): confounding controlled outcomes adjusted for pertinent risk factors (two stars). The third is outcome (three stars): assessment of outcome by independent blind assessment (two stars) and statistical tests clearly described (one star). The following scoring algorithms were used to assess the quality of each study: a study receiving 7–10, 5–6, and 0–4 points was considered to have good, fair, and poor quality, respectively. We only included articles of fair to good quality to preserve this systematic review's validity. Finally, articles with a fair to good quality rating were considered high quality and included in the meta-analysis.

### Data synthesis and statistical test

The characteristics of each of the included studies and the main findings were described in a summary table. The extracted data were imported into Stata software version 15 for quantitative statistical analysis. The results of the meta-analysis were graphically displayed by using forest plots. Cochran's *Q* statistic was used to assess the heterogeneity among the included studies, and *I*^2^ statistics were used to quantify the amount of heterogeneity (44). The presence of heterogeneity across included studies was considered when the *p*-value was <0.1 or *I*^2^ > 50%. As substantial heterogeneity was exhibited among studies, a random-effects model was used to determine pooled estimates. To minimize heterogeneity among the primary studies, subgroup analysis was conducted based on study region, study setting, study year, and sample size. The evidence of publication bias was examined using Egger's regression test and a visual inspection of the asymmetry in the funnel plot (45). Egger's test regression was considered to be evidence of publication if a *p*-value was <0.05. Nonparametric trim and fill analysis was carried out using random-effects model analysis in cases where evidence of publication bias was identified (46). A sensitivity analysis was carried out to determine whether a single study affects the overall estimate (47).

## Results

### Search result

**Figure 1:**
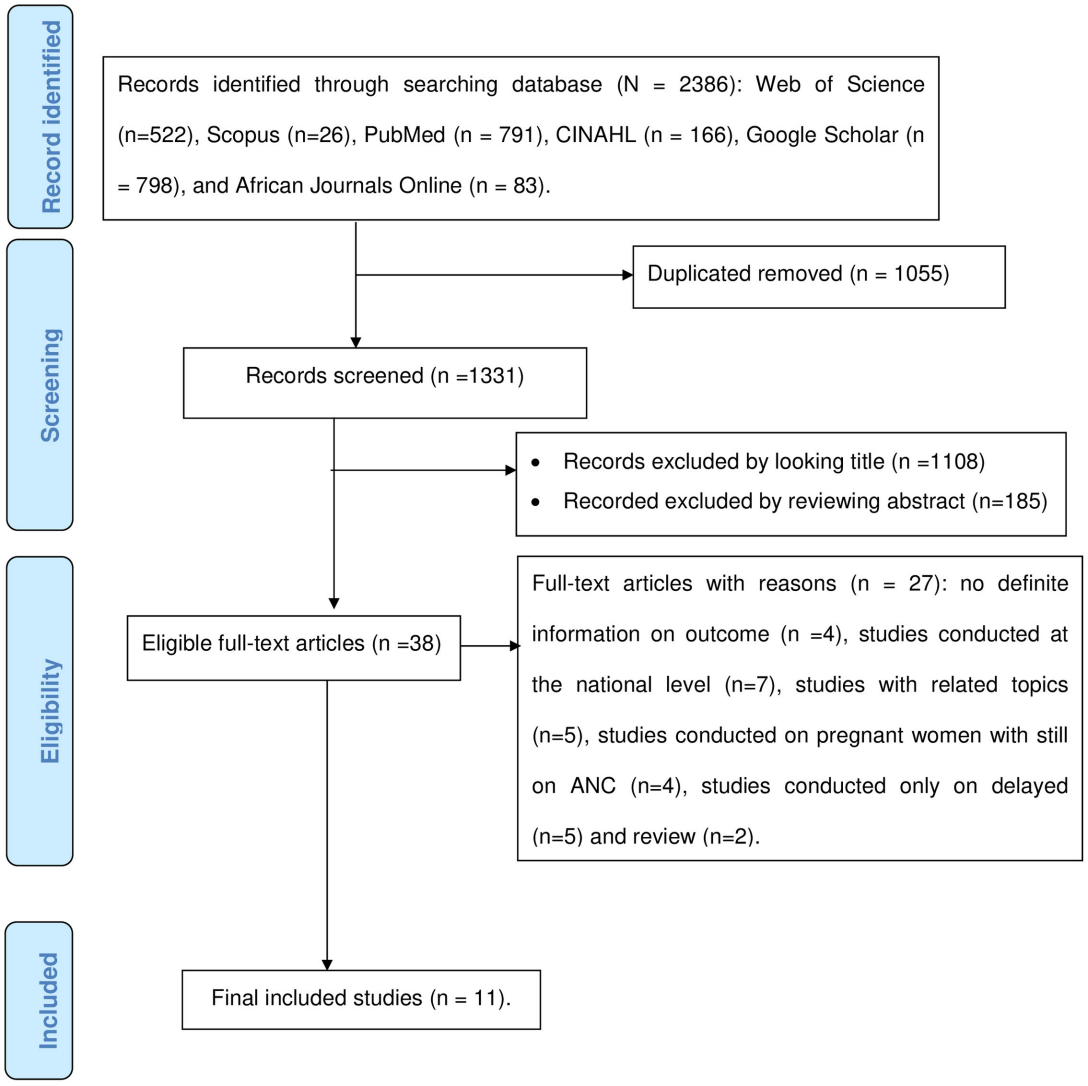
PRISMA flow chart for the selection of studies for systematic review.

Using various international databases and their search algorithms, a total of 2,386 articles were found. A total of 1,331 articles retained after 1,055 duplicates were removed. After two authors (TGW and ZS) screened the remaining articles by examining their titles and abstracts, 1,108 articles with unrelated titles and 185 articles after reviewing their abstracts were excluded. Out of 38 articles that remained for full-text evaluation, 27 were excluded for a variety of reasons, including no definite information on outcome, studies conducted at the national level, studies on pregnant women receiving ANC, studies with related topics, studies that reported the magnitude of delayed ANC only, and review studies. In the end, 11 eligible articles were included in the final meta-analysis (Figure 1).

### Study characteristics

A total of 7,387 study participants were included in this systematic review and meta-analysis; of these, 3,064 had adequate ANC service utilization during the COVID-19 pandemic. All 11 included studies were cross-sectional. All study subjects were reproductive-age women who had already given birth. Five studies were conducted in the Amhara region (48–52). Two studies were conducted in the Oromia region (53, 54). One study was carried out in each of the following regions: Sidama (55), SNNP (56), Dire Dawa (57), and Afar (58). In 2020, six studies were conducted (51–53, 55, 56, 58). Two of the studies (48, 49) were conducted in 2020–2021; the other two studies (54, 57) were conducted in 2021. One study was conducted in 2022 (50). Nine studies were conducted in community-based settings, while two studies were conducted in health institutions. The smallest sample size was 230 (57), and the largest was 1,130 (55). The percentages of minimum and maximum adequate antenatal care were 20.7% (50) and 78.5% (51), respectively. Response rates of the studies ranged from 94.2% to 100% (Table 3).

**Table 3:** Characteristics of the included studies.

**Authors, publication year**	**Study period**	**Study region**	**Study setting**	**Study subjects**	**Objective of study**	**Sampling method**	**Sample size**	**Adequate ANC**	
								**No**.	**%**
Gelagay et al. (48)	2020–2021	Amhara	CB	Reproductive age women	Inadequate ANC	Census method	871	246	28.2
Belay et al. (49)	2020–2021	Amhara	CB	Reproductive age women	Optimal ANC	Random sampling	434	256	59.3
Yoseph et al. (55)	2020	Sidama	CB	Reproductive age women	ANC utilization	Random sampling	1,130	319	28.2
Belay et al (56)	2020	SNNP	CB	Reproductive age women	ANC utilization	Random sampling	978	548	56
Worku et al. (57)	2021	Dire Dawa	HIB	Reproductive age women	ANC dropout	Random sampling	230	144	62.6
Turi et al. (53)	2020	Oromia	CB	Reproductive age women	ANC utilization	Random sampling	827	211	25.5
Gedef et al. (50)	2022	Amhara	CB	Reproductive age women	ANC utilization	Random sampling	593	123	20.7
Urmale Mare et al. (58)	2020	Afar	CB	Reproductive age women	Recommended ANC utilization	Random sampling	703	302	43
Deressa et al. (54)	2021	Oromia	HIB	Reproductive age women	ANC utilization	Random sampling	420	288	68.6
Tizazu et al. (51)	2020	Amhara	CB	Reproductive age women	Minimum four ANC visits	Random sampling	390	306	78.5
Hailemariam et al. (52)	2020	Amhara	CB	Reproductive age women	Optimum ANC utilization	Random sampling	811	321	39.6

CB, community based; HIB, health institution based.

### Quality (risk of bias) assessment

The qualities of the included studies were evaluated using Newcastle-Ottawa appraisal check lists. Included studies were cross-sectional. The quality of all included studies ranged from 7 (70%) to 10 (100%). Two studies were scored 7 out of 10, and three studies were scored 8 out of 10. The remaining six studies scored 10 out of 10. All included studies had good quality (Supplementary File S4).

### The pooled estimate of adequate antenatal care (ANC) service utilization after COVID-19 pandemic

**Figure 2:**
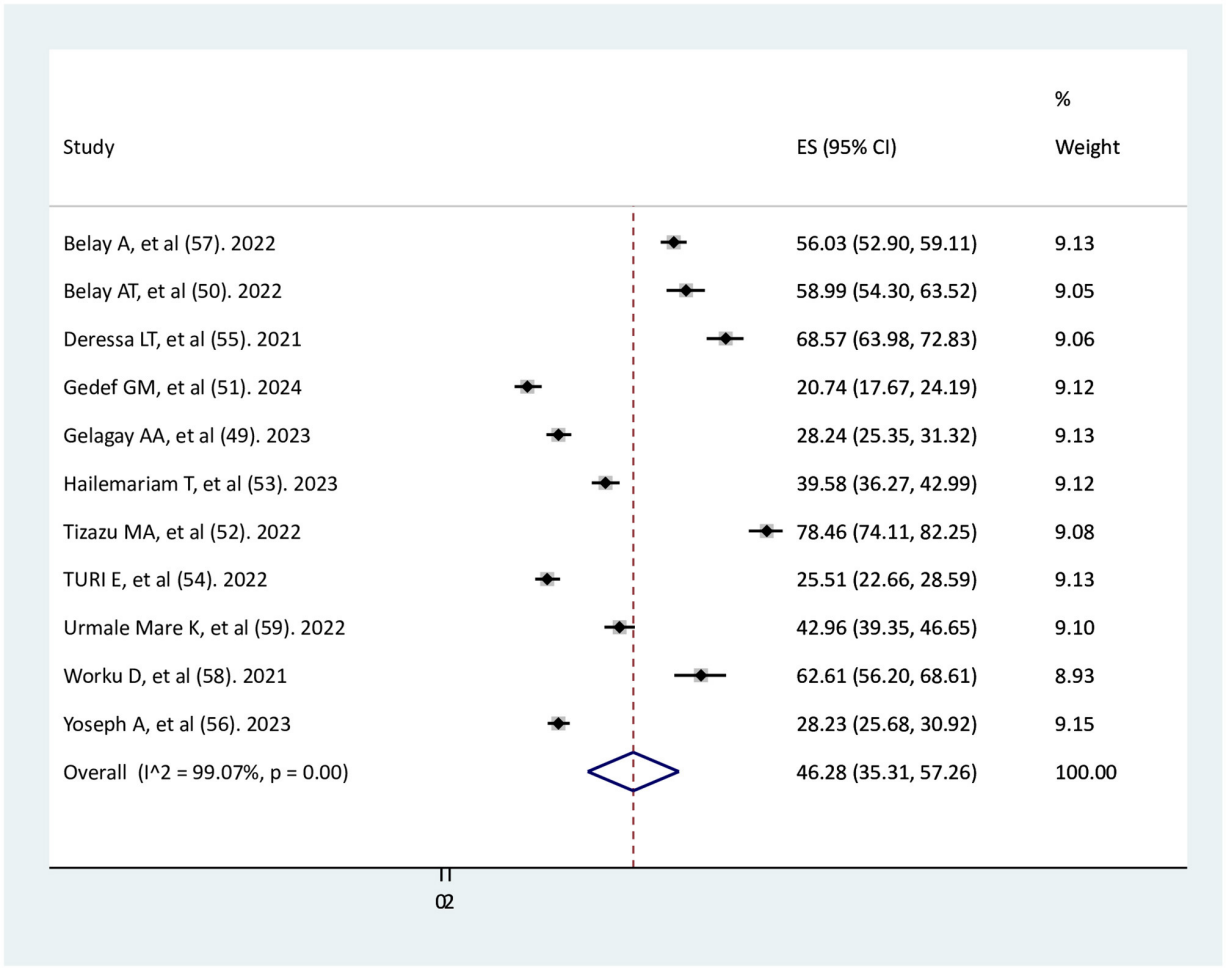
Forest plot of the pooled estimate of adequate ANC service utilization after the onset of COVID-19 pandemic.

Using the random-effect model, the overall pooled estimate of adequate ANC service utilization after COVID-19 pandemic was 46.28% (95% CI: 35.32–57.26%). There was a substantial amount of heterogeneity among the included studies (*I*^2^ = 99.07%, *p* < 0.001; Figure 2).

### Subgroup analysis

In the subgroup analysis by region, the highest percentages of adequate ANC service utilization during the COVID-19 pandemic were 63% (95% CI: 56%−69%) and 56% (95% CI: 53%−59%) in Dire Dawa and SNNP, respectively. In the Amhara region, adequate ANC service utilization during the COVID-19 pandemic was 45% (95% CI: 26%−65%) with substantial heterogeneity (*I*^2^ = 99.1%, *p* < 0.001). Adequate ANC service utilization was 67% (95% CI: 63%−70%) in studies conducted in health institutions with no heterogeneity and 42 % (95%CI: 31%−53%) in studies conducted in communities with heterogeneity (*I*^2^ = 99.1%, *p* < 0.001). In 2020, 2021, and 2022, the correspondent rates of adequate ANC service utilization were 45% (95% CI: 31%−60%), 67% (95% CI: 63%−70%), and 21% (95% CI: 18%−24%), respectively. The utilization of adequate ANC services during the 2020–2021 study periods was 37 % (95% CI: 35%−40%). During the 2020 study period, a significant level of heterogeneity was noted (*I*^2^ = 99.2%, *p* < 0.001). In the sample size of less than 422, adequate ANC service utilization was 70% (95% CI: 61%−79%) with the absence of heterogeneity. Utilization of adequate ANC service was 38% (95% CI: 29%−47%) in the sample size of ≥422 with high heterogeneity (Table 4).

**Table 4:** Subgroup analysis.

**Variables**	**Categories**	**Included studies**	**Sample size**	**Adequate ANC (95% CI)**	***I*^2^, *p*-value**
Region	Amhara	5	3,099	45% (26%−65%)	99.3%, <0.001
	Oromia	2	1,247	39% (36%−41%)	–
	SNNP	1	978	56% (53%−59%)	–
	Dire Dawa	1	230	63% (56%−69%)	–
	Sidama	1	1,130	28% (26%−31%)	–
	Afar	1	703	43% (39%−47%)	–
Study settings	Health institutions based	2	650	67% (63%−70%)	–
	Community based	9	6737	42% (31%−53%)	99.1%, <0.001
Study period	2020	6	4,839	45% (31%−60%)	99.2%, <0.001
	2020–2021	2	1,305	37% (35%−40%)	–
	2021	2	650	67% (63%−70%)	–
	2022	1	593	21% (18%−24%)	–
Sample size	<422	3	1,040	70% (61%−79%)	–
	≥422	8	6,347	38% (29%−47%)	98.3%, <0.001

### Publication bias

**Figure 3:**
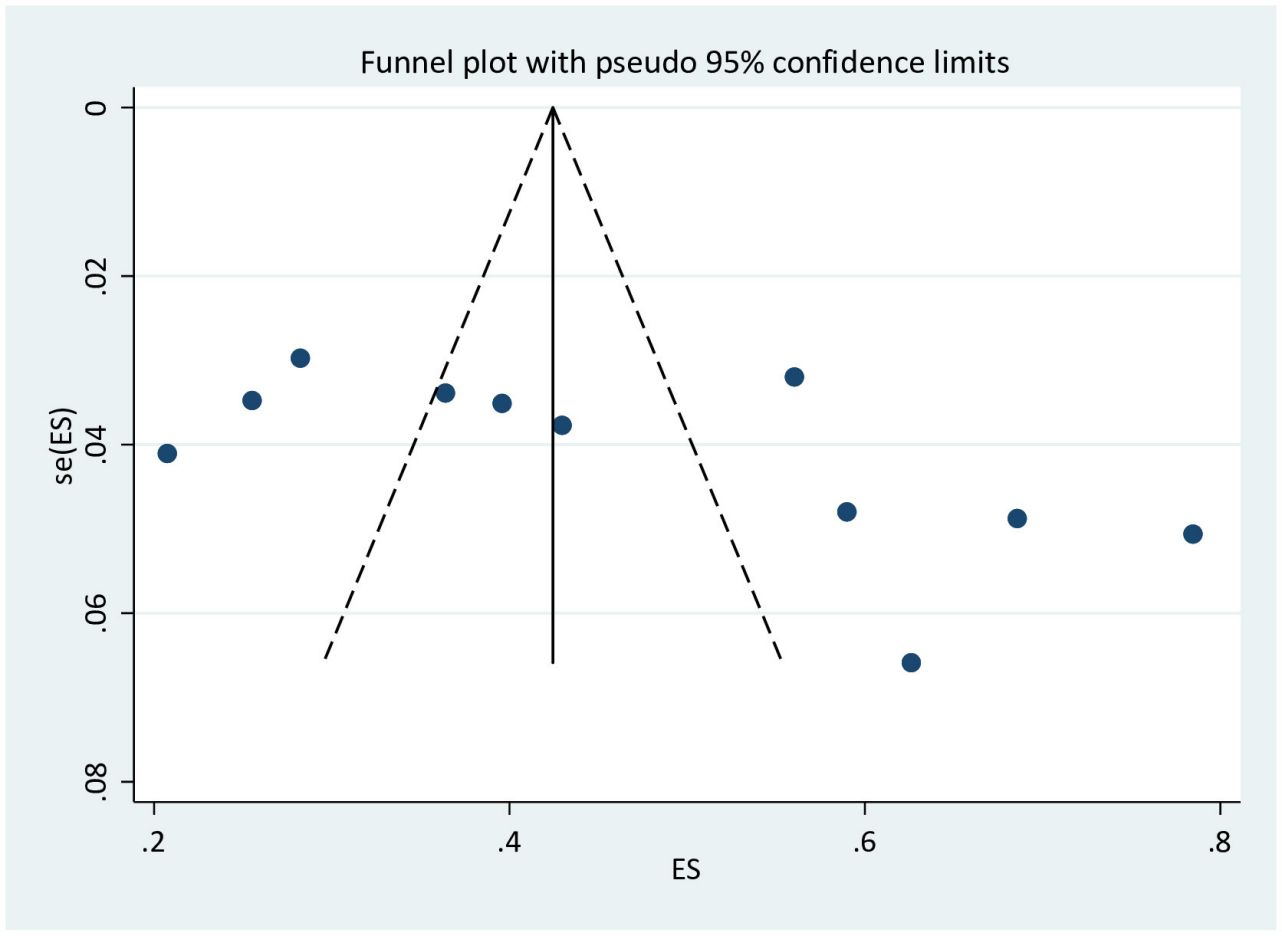
Funnel plot to assess publication bias for adequate ANC utilization.

The result of Egger's test [bias = 12.1 (95% CI: −0.08–24.4), *p* = 0.051] showed the absence of publication bias, but the asymmetric distribution of studies in the funnel plot indicated the existence of publication bias (Figure 3).

### Sensitivity analysis

**Figure 4:**
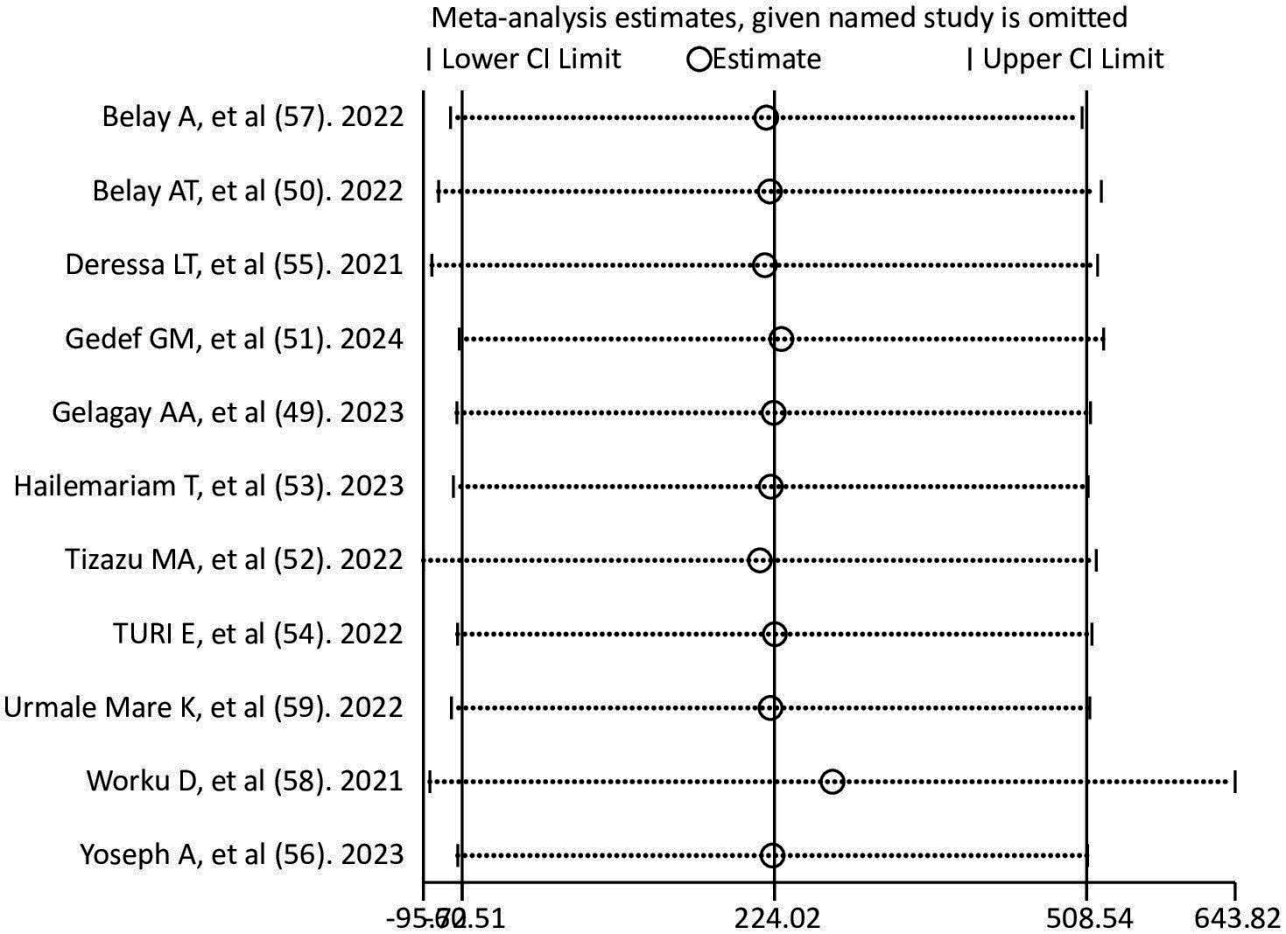
Sensitivity analysis for a pooled estimate of adequate ANC utilization after the onset of the COVID-19 pandemic.

A sensitivity analysis with a random effects model was done to examine any outliers on the overall pooled estimate of adequate ANC service utilization during COVID-19 pandemic. It showed that there was no single study that influenced the overall pooled estimate of adequate ANC service utilization. The results of the sensitivity analysis were fairly stable (Figure 4).

### Associated risk factors for adequate antenatal care (ANC) service utilization

In this study, planned pregnancy, wanted pregnancy, and early ANC visits were eligible factors to conduct a meta-analysis. The results of the meta-analysis showed that early ANC visits had a significant positive association with adequate ANC service utilization; however, planned and wanted pregnancy did not show a significant association with adequate ANC service utilization.

### The association between early ANC visits and adequate ANC service utilization

**Figure 5:**
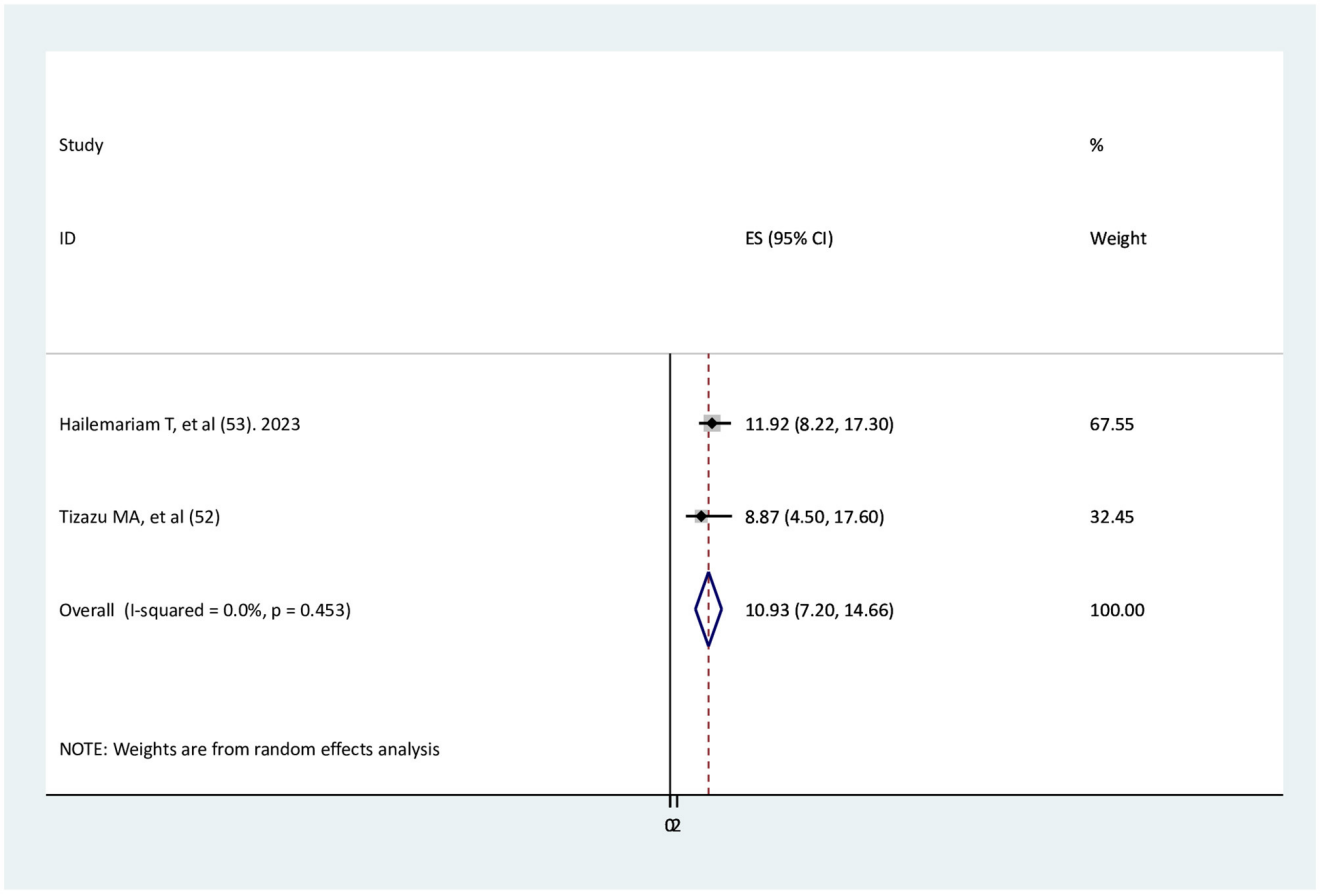
The association between early ANC visits and adequate ANC utilization.

Two studies (51, 52) were found to determine the association between early ANC visits and adequate ANC service utilization. The findings of both studies indicated a significant association with adequate ANC service utilization. The results of the current meta-analysis revealed that pregnant women who visit ANC services early were 10.9 times more likely to have adequate ANC than those without early ANC visits (AOR = 10.93, 95% CI: 7.2–14.66). There is no heterogeneity between studies (Figure 5).

### The association of planned pregnancy with adequate ANC service utilization

**Figure 6:**
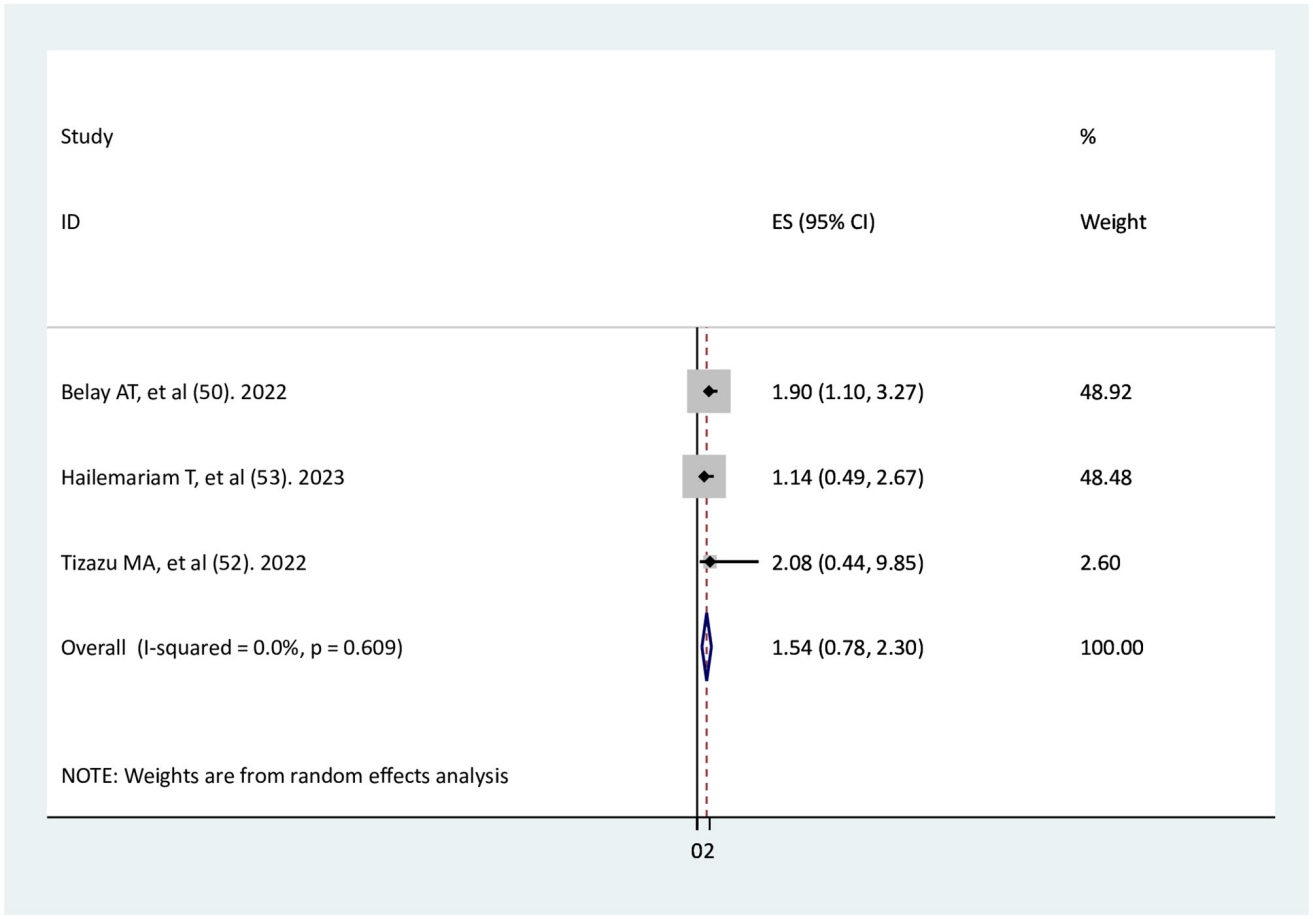
The association of planned pregnancy with adequate ANC service utilization.

Three studies (49, 51, 52) were identified to investigate the relationship between planned pregnancy and adequate ANC service utilization. The findings of the two primary studies (51, 52) indicated that planned pregnancy did not have a significant association with adequate ANC, while one primary study found a significant association (49). The result of the meta-analysis showed no significant association between planned pregnancy and adequate ANC service utilization (AOR = 1.54, 95% CI: 0.78–2.3), with no heterogeneity among the studies (Figure 6).

### The association between wanted pregnancy and adequate ANC service utilization

**Figure 7:**
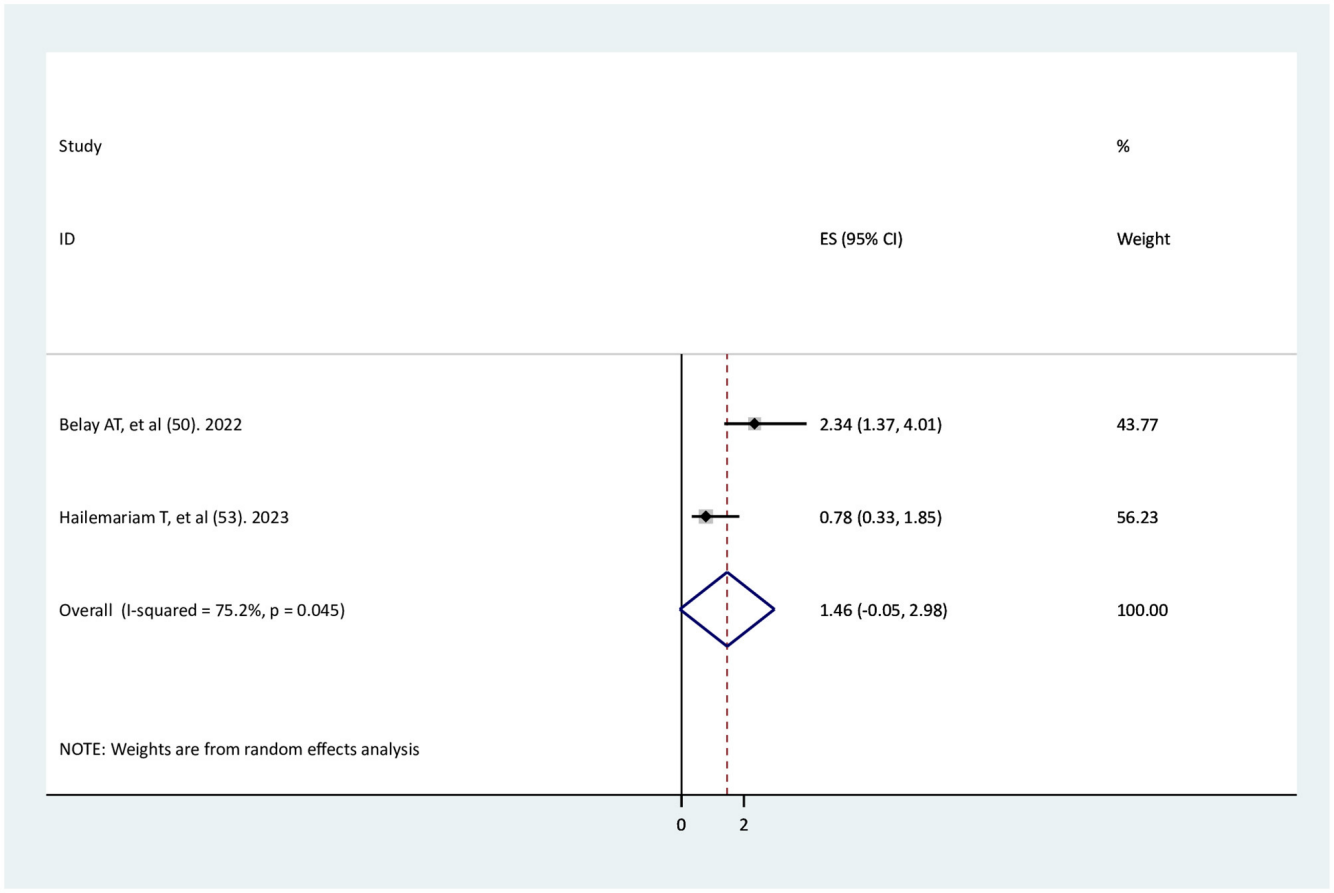
The association between wanted pregnancy and adequate ANC service utilization.

Two studies (49, 52) were used to determine the association between wanted pregnancy and adequate ANC service utilization. One study had a significant association (49), whereas the other one did not have a significant association (52). The findings of this systematic review and meta-analysis revealed that there was no significant association between wanted pregnancy and adequate ANC service utilization (AOR = 1.46, 95% CI: −0.05–2.98), with high significant heterogeneity between studies (*I*^2^ = 75.2%, *p* < 0.001; Figure 7).

## Discussion

In Ethiopia, ANC service utilization after the onset of COVID-19 pandemic was reduced at the national and regional levels (23, 26). In reality, there is no reason to worry about maternal and child health services being overshadowed by the COVID-19 pandemic (38). Improving health system resilience through integrated healthcare service delivery was a potential strategy during the outbreak of the disease (37), but the health systems at the international and national levels collapsed during the COVID-19 pandemic. The delivery of health services, including ANC service, was interrupted and closed in Ethiopia during the COVID-19 pandemic. Therefore, this systematic review and meta-analysis aimed to determine the magnitude of adequate ANC service utilization and its associated risk factors in Ethiopia during the COVID-19 pandemic. This study aims to restore primary healthcare service delivery (2–4) affected by COVID in Ethiopia and improve maternal and newborn health (1).

In this systematic review and meta-analysis, the overall pooled estimate of adequate ANC service utilization after the onset of COVID-19 pandemic in Ethiopia was 46.28%. This result is almost identical to those of studies in Philippines (15) and Uganda (21). This similarity may be due to the worldwide stay-at-home policy (28) and the government's COVID-19 travel restrictions (16) following the WHO's COVID-19 pandemic declaration. Similarly, the current study's magnitude of adequate ANC was comparable with that of Rwanda (20), the mini-EDHS 2019 study (24) and a district-level study conducted between 2019 and 2020 in Ethiopia (25). The current magnitude is also in line with the results of another study conducted in Georgia, which found that the average number of prenatal care visits was about the same before and after the COVID-19 pandemic (18). Studies conducted in Sub-Saharan countries also found similar evidence of a smaller reduction of ANC in 2020 compared to 2019 (17) and a comparable number of ANC visits before the pandemic (2019) and after the pandemic (2020) (19).

However, utilization of adequate ANC service in this meta-analysis was higher than studies conducted in Indonesia (13), Chiapas (16), and Kenya (22). This discrepancy may be due to differences in the severity of COVID-19 in different countries, particularly the occurrence of several critically ill COVID-19 patients (9) in these countries. This could make pregnant women anxious and fearful of contracting COVID-19, as well as vulnerable (28), which could limit their ability to receive adequate ANC services (10).

The pooled estimate of adequate ANC service utilization in this meta-analysis was lower than that in studies conducted in east Africa from 2010 to 2018 (20). This could be attributed to the COVID-19 pandemic's effects, including transportation problems (24, 26, 28), fear of infection, and the implementation of containment measures like social distance and community restrictions (29, 31). Furthermore, the magnitude of adequate ANC in the present study was less than in the previous studies conducted during COVID-19 in Ethiopia (26, 27). The present study's definition of the outcome, which requires that at least four ANC visits are necessary for adequate outcomes, differs from previous studies that defined ANC service utilization broadly and did not put this restriction on at least four ANC utilizations, which could also explain the difference. In general, healthcare service delivery is affected by the COVID-19 pandemic globally (5, 6), such as temporary closure of services (7, 8), and hospitals and healthcare providers are facing challenges in handling a high number of critically ill COVID-19 patients and meeting the need for infection control (9). This has made it extremely difficult for pregnant women to receive prenatal care (10), despite different studies suggesting that women's ANC should be prioritized during the COVID-19 pandemic to safeguard both mother and child health (11, 38, 39).

In the subgroup analysis, the highest percentage of adequate ANC service utilization during the COVID-19 pandemic was observed in Dire Dawa at 63%. This was higher than a previous study that found that ANC utilization during COVID-19 in Dire Dawa was 43.8% (26). Furthermore, the subgroup analysis conducted in this meta-analysis revealed that 45%, 39%, 56%, 28%, and 43% of the Amhara, Oromia, Sidama, SNNP, and Afar regions, respectively, had adequate ANC service utilization. All of these levels of adequate ANC service utilization were lower than those found in the corresponding regions of a prior study (26). There was only one study included in the current systematic review and meta-analysis for every region except the Amhara region. This led to a small sample size, which made it challenging to determine outcomes accurately and allowed chance to play an important role. The subgroup analysis in the Amhara region was 45%, which is comparable with the overall pooled estimate of adequate ANC service utilization in the current meta-analysis. This indicated that most of the studies included in this meta-analysis were conducted in the Amhara region. In studies conducted in health institutions, 67% of women had adequate ANC service utilization, whereas 42% of women had adequate ANC service utilization in community-based studies. This indicates that there are women who still live in the community that are unable to access ANC services. This revealed that studies conducted at health institutions overestimated outcomes, leading to inadequate community representation, a result of selection bias. In 2020, 2021, and 2022, the percentages of adequate ANC service utilization were 45%, 67%, and 21%, respectively. In 2020–2021, adequate ANC service utilization was 37%. This indicates that adequate ANC service utilization is decreasing annually in Ethiopia. In the sample size of <422, the magnitude of adequate ANC service utilization was 70%; on the other hand, in the sample size of ≥422, the magnitude of adequate ANC service utilization was 38%. The inclusion of a small number of studies that led to a high-role chance can be attributed to the high magnitude of adequate ANC service utilization in a sample size of <422.

Pregnant women who visited ANC services earlier were nearly 11 times more likely to have adequate ANC utilization than pregnant women who did not attend early ANC visits. Women with wanted and planned pregnancies were not significantly associated with adequate ANC service utilization. This could be due to the existence of a few studies to determine the association, which can be a cause for being unable to determine the association accurately.

### The limitation and strength of the study

All of the included studies were cross-sectional, so exposure and disease status among individuals assessed at the same time could not clearly determine the temporal association of the factors. The findings of a cross-sectional study can be affected by recall bias and the non-response rate. A few regions in Ethiopia were not represented in this study, which hindered the generalization of the findings to all regions of Ethiopia. In the current study, adequate ANC was considered based on the quantity of ANC (at least four numbers of ANC), but it did not include the quality of ANC. The majority of the pooled odd ratios were computed from a small number of studies, such as two or three studies, which may not provide enough evidence to establish the casual relationship, although it is possible to synthesize meta-analyses utilizing these studies. A high level of heterogeneity was observed in some subgroup analyses of the Amhara region, a community-based study, the study year 2020, and a sample size >420. Variations in the socio-economic and cultural backgrounds of study participants may cause this significant heterogeneity, as these factors affected the utilization of health services. Moreover, the inaccessibility of health service delivery and variations in study methodology were other contributing factors for heterogeneity. On the other hand, in most of the regions, study years, institutional-based studies, and sample sizes less than 420, there is no heterogeneity due to the inclusion of a single or two studies in the subgroup analysis, making it impossible to detect the outcome accurately. The Egger test showed no plication bias, but inspection of the funnel plot indicated an asymmetric distribution of the included studies. The asymmetric nature of the funnel plot, which only depends on effect size and standard error, could be due to variations in the methodology of the included studies, inadequate analysis, and the role of chance. However, the quantitatively pooled adequate ANC service utilization during the COVID-19 pandemic in Ethiopia was extensively addressed in this systematic review. Policymakers, health programmers, and other stakeholders can use the crucial evidence provided by this systematic review and meta-analysis to expand efforts aimed at increasing the uptake of adequate ANC service utilization. Recognizing the impact of the COVID-19 pandemic on adequate ANC service utilization could help establish appropriate strategies and interventions for the improvement of adequate ANC service utilization.

## Conclusion

The pooled estimate of adequate ANC service utilization after the onset of COVID-19 pandemic in Ethiopia was below half the percentage (46.28%). The subgroup analysis by the study year indicated that adequate ANC service utilization was decreasing year over year in Ethiopia. Pregnant women who visited their ANC earlier had higher adequate ANC service utilization than those who did not visit their ANC earlier. Thus, awareness of reproductive-age women focusing on early ANC visits is very important. Furthermore, more research should be done in the future in order to fully investigate factors influencing ANC coverage.

## Data Availability

The original contributions presented in the study are included in the article/Supplementary material, further inquiries can be directed to the corresponding author.

## Supplementary material

Supplementary File S1 Checklist of Preferred Reporting Items for Systematic Reviews and Meta-Analysis (PRISMA 2020).

Supplementary File S2 Comprehensive search strategy for adequate ANC.

Supplementary File S3 Extracted data for adequate ANC.

Supplementary File S4 The quality of included studies.
