# Supplementary material for: Adequate antenatal care service utilizations after the onset of COVID-19 pandemic in Ethiopia: a systematic review and meta-analysis
Source: Front Public Health. 2024 Nov 15;12:1395190. doi: 10.3389/fpubh.2024.1395190 (PMC11605392; doi:10.3389/fpubh.2024.1395190)
Supplement: Supplementary File S2 — Comprehensive search strategy for adequate ANC. [file Table_2.DOCX]

S2 File. Comprehensive search strategy for adequate antenatal care

| Databases | Key search terms or phrases |
| --- | --- |
| **Web of Science** | "Antenatal care utilization" Ethiopia |
| Restricted with | English language, articles and publication years between 01/01/2020 and 08/08/2024 |
| Total articles | 522 |
| **Scopus** | "Antenatal care utilization" Ethiopia |
| Restricted with | English language, research articles, and publication years of 2020–2024 |
| Total articles | 26 |
| **PubMed** | ((((Adequate) OR (Optimal) AND ((2020/1/1:2024/02/22[pdat]) AND (english[Filter]))) OR ((((("Minimum recommended") AND ("World Health Organization"[MeSH Terms])) OR ("World Health Organization")) OR ("WHO")) OR ("Organization, World Health") AND ((2020/1/1:2024/02/22[pdat]) AND (english[Filter])))) AND ((((("Prenatal Care"[MeSH Terms]) OR ("Prenatal Care")) OR ("Care, Prenatal")) OR ("Antenatal Care")) OR ("Care, Antenatal") AND ((2020/1/1:2024/02/22[pdat]) AND (english[Filter])))) AND ((Ethiopia[MeSH Terms]) OR (Ethiopia) AND ((2020/1/1:2024/02/22[pdat]) AND (english[Filter]))) |
| Restricted with | English language, free full text and publication date from 01/01/2020 to 02/22/2024 |
| Total articles | 791 |
| **CINAHL** | “adequate “antenatal care service utilization” Ethiopia” |
| Restricted with | full text, English language, and publication date from 01/01/2020 to 02/22/2024 |
| Total articles | 166 |
| **Google scholar** | "Adequate” OR "Optimal" “Antenatal care utilization” "Ethiopia" |
| Restricted with | English language and Publication date 2020 to 2024 |
| Total articles | 798 |
| **African Journals Online** | Adequate "antenatal care service utilization" Ethiopia |
| Total articles | 83 |
| Total articles retrieved from all databases | 2386 |
