# Supplementary material for: Adequate antenatal care service utilizations after the onset of COVID-19 pandemic in Ethiopia: a systematic review and meta-analysis
Source: Front Public Health. 2024 Nov 15;12:1395190. doi: 10.3389/fpubh.2024.1395190 (PMC11605392; doi:10.3389/fpubh.2024.1395190)
Supplement: Supplementary File S3 — Extracted data for adequate ANC. [file Table_3.DOC]

S3 File Extracted data for adequate ANC service utilization

| ID | **Authors, publication year** | Study period | Study region | Study setting | Sample size | Adequate ANC | Percent  (%) | Response  Rate |
| --- | --- | --- | --- | --- | --- | --- | --- | --- |
| 1 | Gelagay AA, et al. (49), 2023 | 2020-2021 | Amhara | CB | 871 | 246 | 28.2 | 100% |
| 2 | Belay AT, et al. (50), 2022 | 2020-2021 | Amhara | CB | 434 | 256 | 59.3 | 100% |
| 3 | Yoseph A, et al. (56), 2023 | 2020 | Sidama | CB | 1130 | 319 | 28.2 | 99.1% |
| 4 | Belay A, et al. (57), 2022 | 2020 | SNNP | CB | 978 | 548 | 56 | 100% |
| 5 | Worku D, et al. (58), 2021 | 2021 | Dire Dawa | HIB | 230 | 144 | 62.6 | 100% |
| 6 | TURI E, et al. (54), 2022 | 2020 | Oromia | CB | 827 | 211 | 25.5 | 97.9% |
| 7 | Gedef GM, et al. (51), 2024 | 2022 | Amhara | CB | 593 | 123 | 20.7 | 100% |
| 8 | Urmale Mare K, et al.(59), 2022 | 2020 | Afar | CB | 703 | 302 | 43 | 94.2% |
| 9 | Deressa LT, et al. (55), 2021 | 2021 | Oromia | HIB | 420 | 288 | 68.6 | 100% |
| 10 | Tizazu MA, et al. (52), 2022 | 2020 | Amhara | CB | 390 | 306 | 78.5 | 99.2% |
| 11 | Hailemariam T, et al. (53), 2023 | 2020 | Amhara | CB | 811 | 321 | 39.6 | 100% |

ANC: antenatal care, SNNP: southern nation nationality of people, CB: community based, HIB: health institution based
