# Supplementary material for: Adequate antenatal care service utilizations after the onset of COVID-19 pandemic in Ethiopia: a systematic review and meta-analysis
Source: Front Public Health. 2024 Nov 15;12:1395190. doi: 10.3389/fpubh.2024.1395190 (PMC11605392; doi:10.3389/fpubh.2024.1395190)
Supplement: Supplementary File S4 — The quality of included studies. [file Table_4.docx]

| ID | Authors, Publication year | Reviewers | Selection | | | | Comparability | Outcome | | Total score | Quality |
| --- | --- | --- | --- | --- | --- | --- | --- | --- | --- | --- | --- |
|  |  |  | 1 | 2 | 3 | 4 |  | 1 | 2 |  |  |
| 1 | Gelagay AA, et al. (49), 2023 | TGW | * | * | ⁎ | ⁎⁎ | ⁎⁎ | ⁎⁎ | ⁎ | 10/10=100% | Good quality |
|  |  | ZS | * | * | ⁎ | ⁎⁎ | ⁎⁎ | ⁎⁎ | ⁎ | 10/10=100% |  |
|  |  | Average score | 1 | 1 | 1 | 2 | 2 | 2 | 1 | 10/10=100% |  |
| 2 | Belay AT, et al. (50), 2022 | TGW | * | * | ⁎ | ⁎⁎ | ⁎⁎ | ⁎⁎ | ⁎ | 10/10=100% | Good quality |
|  |  | ZS | * | * | ⁎ | ⁎⁎ | ⁎⁎ | ⁎⁎ | ⁎ | 10/10=100% |  |
|  |  | Average score | 1 | 1 | 1 | 2 | 2 | 2 | 1 | 10/10=100% |  |
| 3 | Yoseph A, et al. (56), 2023 | TGW | * | * | ⁎ | ⁎⁎ | 0 | ⁎⁎ | ⁎ | 8/10=80% | Good quality |
|  |  | ZS | * | * | ⁎ | ⁎⁎ | 0 | ⁎⁎ | ⁎ | 8/10=80% |  |
|  |  | Average score | 1 | 1 | 1 | 2 | 0 | 2 | 1 | 8/10=80% |  |
| 4 | Belay A, et al. (57), 2022 | TGW | ⁎ | ⁎ | ⁎ | ⁎⁎ | 0 | ** | * | 8/10=80% | Good quality |
|  |  | ZS | ⁎ | ⁎ | ⁎ | ⁎⁎ | 0 | ⁎* | * | 8/10=80% |  |
|  |  | Average score | 1 | 1 | 1 | 2 | 0 | 1 | 1 | 8/10=80% |  |
| 5 | Worku D, et al. (58), 2021 | TGW | ⁎ | ⁎ | ⁎ | ⁎⁎ | ⁎⁎ | ** | ⁎ | 10/10=100% | Good quality |
|  |  | ZS | ⁎ | ⁎ | ⁎ | ⁎⁎ | ⁎⁎ | ** | ⁎ | 10/10=100% |  |
|  |  | Average score | 1 | 1 | 1 | 2 | 2 | 2 | 1 | 10/10=100% |  |
| 6 | TURI E, et al. (54), 2022 | TGW | * | * | * | ** | 0 | ** | * | 8/10=80% | Good quality |
|  |  | ZS | * | * | * | ** | 0 | ** | * | 8/10=80% |  |
|  |  | Average score | 1 | 1 | 1 | 2 | 0 | 2 | 1 | 8/10=80% |  |
| 7 | Gedef GM, et al. (51), 2024 | TGW | * | * | * | ** | 0 | * | * | 7/10=70% | Good quality |
|  |  | ZS | * | * | * | ** | 0 | * | * | 7/10=70% |  |
|  |  | Average score | 1 | 1 | 1 | 2 | 0 | 1 | 1 | 7/10=70% |  |
| 8 | Urmale Mare K, et al.(59), 2022 | TGW | * | * | * | ** | ** | ** | * | 10/10=100% | Good quality |
|  |  | ZS | * | * | * | ** | ** | ** | * | 10/10=100% |  |
|  |  | Average score | 1 | 1 | 1 | 2 | 2 | 2 | 1 | 10/10=100% |  |
| 9 | Deressa LT, et al. (55), 2021 | TGW | * | * | * | ** | 0 | * | * | 7/10=70% | Good quality |
|  |  | ZS | * | * | * | ** | 0 | * | * | 7/10=70% |  |
|  |  | Average score | 1 | 1 | 1 | 2 | 0 | 1 | 1 | 7/10=70% |  |
| 10 | Tizazu MA, et al. (52), 2022 | TGW | * | * | * | ** | ** | ** | * | 10/10=100% | Good quality |
|  |  | ZS | * | * | * | ** | ** | ** | * | 10/10=100% |  |
|  |  | Average score | 1 | 1 | 1 | 2 | 2 | 2 | 1 | 10/10=100% |  |
| 11 | Hailemariam T, et al. (53), 2023 | TGW | * | * | ⁎ | ⁎⁎ | ⁎⁎ | ⁎⁎ | * | 10/10=100% | Good quality |
|  |  | ZS | * | * | ⁎ | ⁎⁎ | ⁎⁎ | ⁎⁎ | ⁎ | 10/10=100% |  |
|  |  | Average score | 1 | 1 | 1 | 2 | 2 | 2 | 1 | 10/10=100% |  |

# S4 File displays the average quality of the included studies’ scores evaluated by Temesgen Gebeyehu Wondmeneh (TGW) and Zelalem Solomon (ZS) using the Newcastle-Ottawa Scale Appraisal Checklist.

The three domains of categories are listed below, along with their subsections and rating stars.

## Selection:

1. Representativeness of the sample:
   1. Truly representative of the average in the target population. * (all subjects or random sampling)
   2. Somewhat representative of the average in the target group. * (non-random sampling)
   3. Selected group of users/convenience sample.
   4. No description of the derivation of the included subjects.
2. Sample size:
   1. Justified and satisfactory (including sample size calculation). *
   2. Not justified.
   3. No information provided
3. Non-respondents:
   1. Proportion of target sample recruited attains pre-specified target or basic summary of non-respondent characteristics in sampling frame recorded. *
   2. Unsatisfactory recruitment rate, no summary data on non-respondents.
   3. No information provided
4. Ascertainment of the exposure (risk factor):
   1. Vaccine records/vaccine registry/clinic registers/hospital records only. **
   2. Parental or personal recall and vaccine/hospital records.*
   3. Parental/personal recall only.

**Comparability:** (Maximum 2 stars)

1. Comparability of subjects in different outcome groups on the basis of design or analysis. Confounding factors controlled.

1. Data/ results adjusted for relevant predictors/risk factors/confounders e.g. age, sex, time since vaccination, etc. **
2. Data/results not adjusted for all relevant confounders/risk factors/information not provided

Assessment of outcome (maximum 3 stars):

- - 1. Independent blind assessment using objective validated laboratory methods. **
    2. Non-blinded assessment using objective validated laboratory methods. **
    3. Used non-standard or non-validated laboratory methods with gold standard. *
    4. No description/non-standard laboratory methods used.
  1. Statistical test:
     1. Statistical test used to analyse the data clearly described, appropriate and measures of association presented including confidence intervals and probability level (p value). *
     2. Statistical test not appropriate, not described or incomplete.

# This scale has been adapted from the Newcastle-Ottawa Quality Assessment Scale for cohort studies to provide quality assessment of cross sectional studies^1^.
